# A global dataset of terrestrial biological nitrogen fixation

**DOI:** 10.1038/s41597-025-05131-4

**Published:** 2025-08-05

**Authors:** Carla R. Reis Ely, Steven S. Perakis, Cory C. Cleveland, Duncan N. L. Menge, Sasha C. Reed, Sarah A. Batterman, Timothy E. Crews, Katherine A. Dynarski, Maga Gei, Michael J. Gundale, Sarah E. Jovan, Sian Kou-Giesbrecht, Mark B. Peoples, Emilio Rodríguez-Caballero, Verity G. Salmon, Fiona M. Soper, Anika P. Staccone, Benton N. Taylor, Bettina Weber, Nina Wurzburger

**Affiliations:** 1 Department of Forest Ecosystems and Society, Oregon State University, Corvallis, OR 97331 USA; 2 Oak Ridge Institute for Science and Education, Oak Ridge, TN 37830 USA; 3 United States Geological Survey, Forest and Rangeland Ecosystem Science Center, Corvallis, OR 97331 USA; 4 Department of Ecosystem and Conservation Sciences, W.A. Franke College of Forestry and Conservation, University of Montana, Missoula, MT 59812 USA; 5 Department of Ecology, Evolution, and Environmental Biology, Columbia University, New York, NY 10027 USA; 6 United States Geological Survey, Southwest Biological Science Center, Moab, UT 84532 USA; 7 Cary Institute of Ecosystem Studies, Millbrook, NY 12545 USA; 8 School of Geography, University of Leeds, Leeds, UK; 9 Smithsonian Tropical Research Institute, Ancon, Panama; 10 The Land Institute, Salina, KS 67401 USA; 11 Association for Tropical Biology and Conservation, Minneapolis, MN 55405 USA; 12 Department of Forest Ecology and Management, Swedish University of Agricultural Sciences, 90183 Umea, Sweden; 13 USDA Forest Service, PNW Research Station, Portland, OR USA; 14 Department of Earth and Environmental Sciences, Dalhousie University, Halifax, NS B3H 4R2 Canada; 15 CSIRO Agriculture and Food, GPO Box 1700, Canberra, ACT 2601 Australia; 16 Centro de Investigación de Colecciones Científicas de la Universidad de Almería (CECOUAL) y Departamento de Agronomía, Universidad de Almería, Almería, Spain; 17 Multiphase Chemistry Department, Max Planck Institute for Chemistry, Mainz, Germany; 18 Environmental Science Division and Climate Change Science Institute, Oak Ridge National Laboratory, Oak Ridge, TN 37830 USA; 19 Department of Biology and School of the Environment, McGill University, Montreal, QC H3A 1B1 Canada; 20 Earthshot Labs, Sebastapol, CA 95472 USA; 21 Department of Organismic and Evolutionary Biology, Harvard University, 1300 Centre St., Roslindale, MA 02131 USA; 22 Division of Plant Sciences, Institute for Biology, University of Graz, Graz, Austria; 23 Odum School of Ecology, University of Georgia, Athens, GA 30602 USA

**Keywords:** Element cycles, Ecosystem ecology

## Abstract

Biological nitrogen fixation (BNF) is the main natural source of new nitrogen inputs in terrestrial ecosystems, supporting terrestrial productivity, carbon uptake, and other Earth system processes. We assembled a comprehensive global dataset of field measurements of BNF in all major N-fixing niches across natural terrestrial biomes derived from the analysis of 376 BNF studies. The dataset comprises 32 variables, including site location, biome type, N-fixing niche, sampling year, quantification method, BNF rate (kg N ha^−1^ y^−1^), the percentage of nitrogen derived from the atmosphere (%N_dfa_), N fixer or N-fixing substrate abundance, BNF rate per unit of N fixer abundance, and species identity. Overall, the dataset combines 1,207 BNF rates for trees, shrubs, herbs, soil, leaf litter, woody litter, dead wood, mosses, lichens, and biocrusts, 152 herb %N_dfa_ values, 1,005 measurements of N fixer or N-fixing substrate abundance, and 762 BNF rates per unit of N fixer abundance for a total of 424 species across 66 countries. This dataset facilitates synthesis, meta-analysis, upscaling, and model benchmarking of BNF fluxes at multiple spatial scales.

## Background & Summary

Nitrogen (N) availability is one of the main factors regulating terrestrial productivity, carbon (C) uptake, and organic matter decomposition in the biosphere^1–3^. Biological N fixation (BNF) is the process whereby atmospheric dinitrogen gas (N_2_) is converted into biologically available N. BNF is carried out by specific prokaryotes that possess the enzyme nitrogenase. N-fixing prokaryotes occupy myriad N-fixing niches, both as free-living bacteria (*e.g*., in soil, litter, and dead wood) and in symbiosis or association with plants or other organisms (*e.g*., trees, shrubs, herbs, mosses, lichens, and biocrusts)^4,5^. BNF represents the primary natural source of new N inputs in most terrestrial ecosystems worldwide, replenishing N losses and supporting new growth^6,7^. However, terrestrial BNF estimates at multiple spatial scales remain considerably uncertain^8–10^. This uncertainty is partly due to the challenge of constraining BNF measurements across the multitude of N-fixing niches within ecosystems^4,5^ and may reflect sampling bias in field BNF studies that favor geographic hotspots where N fixers and N-fixing substrates (*i.e*., the material in which N fixation occurs, for example, dead wood) are abundant^8^. A comprehensive accounting of BNF fluxes by individual N-fixing niches and their abundances is essential for improving accuracy and reducing uncertainty in terrestrial BNF estimates.

**Fig. 1:**
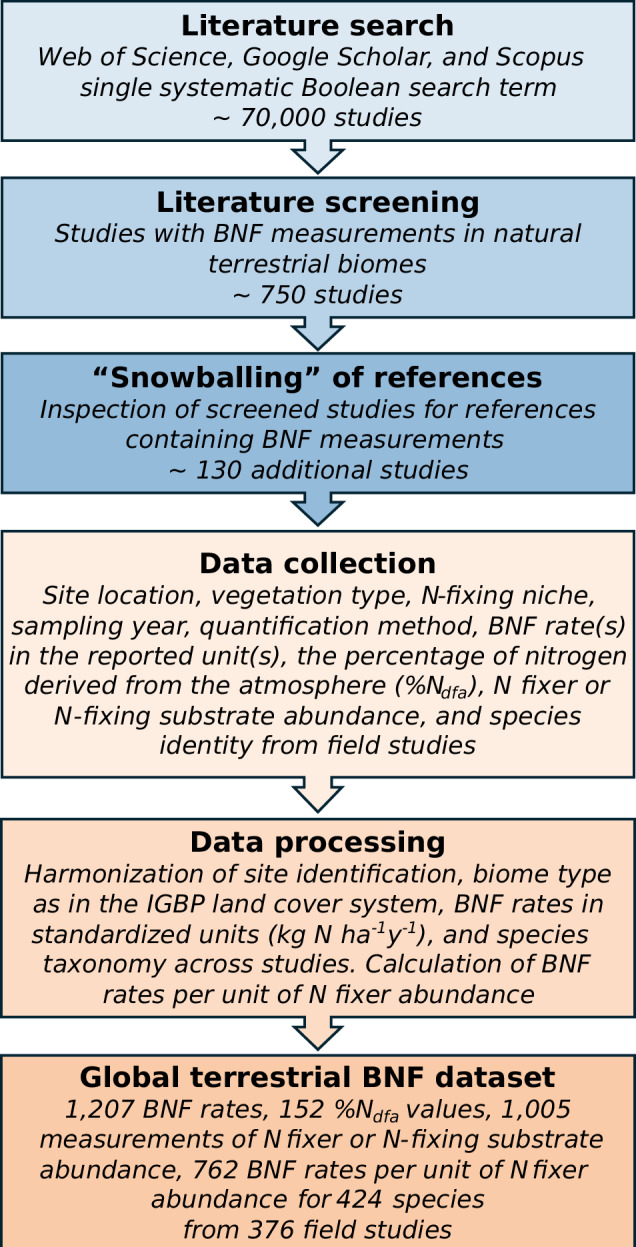
Workflow and systematic approaches for developing the global dataset of biological nitrogen fixation (BNF) in natural terrestrial biomes^11^.

**Fig. 2:**
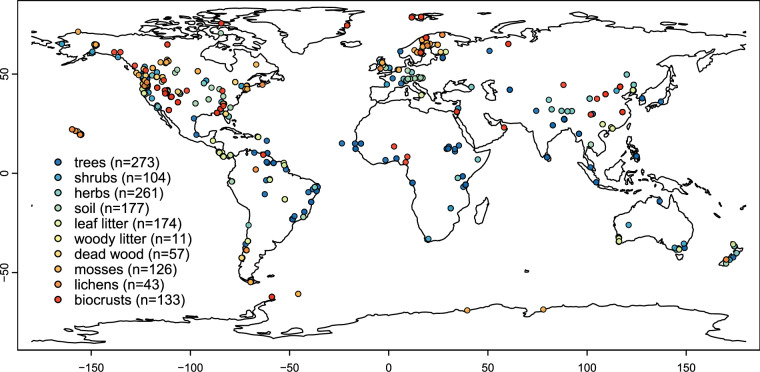
Location of field measurements of BNF in the global BNF dataset in natural terrestrial biomes^11^. Each point represents one BNF rate (kg N ha^−1^ y^−1^) and/or percentage of nitrogen derived from the atmosphere (%N_dfa_) value (n = 1,359) for root-nodulating N-fixing trees, shrubs, or herbs, free-living BNF in soil, leaf litter, woody litter, or dead wood, or BNF associated with mosses, lichens, or biocrusts. Not all points are visible due to overlap. Maps for each niche are in Supplementary Figures S2 to S4.

Here, we assembled a global dataset of field measurements of BNF in all major N-fixing niches across natural terrestrial biomes^11^. Using systematic approaches of literature search and data collection and processing (Fig. 1), we generated a dataset with 32 variables, including site location, the International Geosphere-Biosphere Program (IGBP) land cover class^12^, N-fixing niche (root-nodulating N-fixing trees, shrubs, and herbs, free-living BNF in soil, leaf litter, woody litter, dead wood, and BNF associated with mosses, lichens, and biocrusts), sampling year, quantification method, BNF rate (kg N ha^−1^ y^−1^), the percentage of N derived from the atmosphere (%N_dfa_), N fixer or N-fixing substrate abundance, BNF rate per unit of N fixer abundance, and species identity. The dataset includes 376 BNF studies and combines 1,359 BNF measurements (1,207 BNF rates and 152 %N_dfa_ values) (Fig. 2), 1,005 measurements of N fixer or N-fixing substrate abundance, and 762 BNF rates per unit of N fixer abundance for a total of 424 species across 66 countries. This dataset facilitates synthesis, meta-analysis, upscaling, and model benchmarking of BNF rates, as well as evaluation of the effects of environmental change on BNF fluxes at multiple spatial scales^4,5,8^. To our knowledge, no other dataset with site-level BNF rates in natural terrestrial ecosystems is currently available in repositories or data journals. Global gridded datasets of natural terrestrial BNF fluxes upscaled using the BNF dataset presented here, and spatially explicit abundances of N-fixing niches globally are available at 10.5066/P13THKNR^8,13^.

## Methods

### Literature search

We performed a literature search using a single systematic Boolean search term (*i.e*., using “AND” and “OR” filters) to combine N fixation terms with lists of natural terrestrial biomes, countries, N-fixing taxa, and N-fixing niches (Supplementary Text S1). We used this search term in the software Publish or Perish (https://harzing.com/resources/publish-or-perish) to search for BNF publications in the Web of Science (https://www.webofscience.com), Google Scholar (https://scholar.google.com), and Scopus (https://www.scopus.com) databases. This search resulted in a list with ~ 70,000 references (Fig. 1). We screened studies manually and selected those reporting BNF measurements in natural and semi-natural terrestrial ecosystems. We selected studies in unmanaged and managed ecosystems (*e.g*., silviculture/forestry, rangelands, and agroforestry systems) but excluded studies in grain croplands and forage/livestock-intensive production systems due to more intensive human interference. This screening resulted in ~750 studies that met the criteria for inclusion in the dataset. We also used a snowballing approach wherein we inspected the screened studies to help identify additional publications containing BNF measurements that were not located in the primary search. This snowballing added ~ 130 studies to the dataset. The complete dataset includes ~880 studies conducted in field, growth chamber, greenhouse, shade house, and/or laboratory settings. Here, we describe the subset dataset of 376 studies carried out in the field or other settings simulating field conditions (hereafter “field studies”). The sources included journal articles, dissertations, theses, book chapters, and technical reports published in English, Spanish, French, Chinese, or Russian between 1955 and 2020.

### Data collection

We extracted data manually into a spreadsheet template^5^. Data published as figures were extracted using the software WebPlotDigitizer (https://automeris.io). We recorded BNF measurements from observations and control treatments of field studies. We also recorded BNF measurements for treatments using prescribed fire or grazing in ecosystems where these disturbances occur naturally. For managed ecosystems, we also recorded BNF measurements for treatments using pruning or thinning but excluded data from recent clear-cuts and young plantations (<1 year old) due to more intensive human disturbance. We also excluded data for pure cultures of N-fixing prokaryotes isolated from field samples, as these do not represent N-fixing activity in the presence of a symbiont and/or community.

For each study we recorded the site name, country name (later converted to ISO code), geographic coordinates in decimal degrees, vegetation type as described by authors (later converted to an IGBP land cover class; see Data processing), and N-fixing niche (root-nodulating N-fixing trees, shrubs, and herbs [legume forbs], free-living BNF in soil [mineral and/or organic soil, including humus], litter [all non-woody litter, usually leaf litter], woody litter [dead branches and stems ≤7.5 cm diameter], and dead wood [logs >7.5 cm diameter], and BNF associated with mosses, lichens, and biocrusts). We also recorded the sampling year(s), quantification method(s) (acetylene reduction assay (ARA) [indirect measurement of nitrogenase enzyme activity based on the reduction of acetylene to ethylene by nitrogenase, and the rate of ethylene accumulation], ^15^N_2_ incorporation [measurement of nitrogenase enzyme activity based on the rate of incorporation of ^15^N-labelled N_2_ into tissue], ^15^N natural abundance [measurement of the proportion of N derived from BNF based on the N stable isotopic composition of tissues], ^15^N dilution [measurement of the dilution of an applied ^15^N isotopic label by fixed N], N accumulation [measurement of the difference in N content of a system at two points in time], N accretion [measurement of the difference in N content in a chronosequence to approximate the accumulation in a single site], and mass balance [measurement of the difference between other N fluxes and accrual in a bounded system])^5,14,15^, reported BNF measurements and their units, N fixer or N-fixing substrate abundance from on-site surveys (N fixer relative basal area and/or relative stem density for trees, N fixer percent ground cover for shrubs, herbs, mosses, and biocrusts, and biomass for dead wood), and N fixer family, genus, and species names (Fig. 1 and Table 1). Additionally, we recorded sampled soil depth and bulk density, which we used in soil BNF rate conversions (see Data Processing). Finally, we recorded whether the studied mosses and lichens were epiphytes or located on the ground (“habit”). For field studies relying on methods that require sample incubation (*i.e*., ARA and ^15^N_2_ incorporation), we recorded only BNF measurements taken at temperature and moisture levels within the range experienced in the field at any given time in the year, and in the absence of supplemental C or nutrient additions. We note that the number of significant figures of numeric variables varies among observations, reflecting the precision of reported values.

**Table 1:** Variables in the global BNF dataset in natural terrestrial biomes^11^.

Variable	Type	Unit	Description	*#* Records	*#* Values	Range
record_ID	Integer	NA	Record identification code	1,529	1,529	1; 1,529
site_name	Character	NA	Study site name	1,529	987	NA
country	Character	NA	3-digit ISO country code	1,529	66	NA
lat	Numeric	°	Latitude coordinate	1,529	500^†^	−69; 79
lon	Numeric	°	Longitude coordinate	1,529	500^†^	−164.8262; 175.37
IGBP	Character	NA	International Geosphere-Biosphere Program land cover class	1,528	10	NA
niche	Character	NA	N-fixing niche	1,529	10	NA
ecological_level	Character	NA	If species or community-level data	1,240	2	NA
year	Integer	NA	Sampling year	1,301	57	1952; 2018
method	Integer	NA	Quantification method(s) code	1,359	13	10; 70
BNF_central	Numeric	varies, as reported	Central BNF rate reported	1,188	739	0; 9,432.6
BNF_min	Numeric	varies, as reported	Minimum BNF rate reported	37	29	0; 282
BNF_max	Numeric	varies, as reported	Maximum BNF rate reported	39	34	0.13; 363
BNF_unit	Character	varies, as reported	The unit of reported BNF rate(s)	1,207	48	NA
BNF_final	Numeric	kg N ha^−1^ y^−1^	Final BNF rate in standardized units, based on reported BNF rate(s) and local abundance of N fixers or N-fixing substrate	1,188	718	0; 361.6
BNF_final_type	Integer	NA	Type of final BNF rate code: if originally reported or converted from similar units or other types of BNF data	1,188	6	1; 6
pct_BA	Numeric	%	N fixer relative basal area	269	85	0.8; 100
pct_stems	Numeric	%	N fixer relative stem density	282	66	0.69; 100
pct_cover	Numeric	%	N fixer percent ground cover	401	227	0; 100
AGB	Numeric	Mg ha^−1^	N-fixing substrate aboveground biomass	53	45	0.66; 296.2
BNF_BA	Numeric	kg N ha^−1^ y^−1^ %BA^−1^	BNF rate per unit of N fixer relative basal area	221	173	0; 4.625
BNF_stems	Numeric	kg N ha^−1^ y^−1^ %stems^−1^	BNF rate per unit of N fixer relative stem density	230	178	0; 7.602
BNF_cover	Numeric	kg N ha^−1^ y^−1^ %cover^−1^	BNF rate per unit of N fixer percent ground cover	311	217	0; 11.2
NDFA	Numeric	%	Percentage of nitrogen derived from atmosphere (%N_dfa_)	152	84	0.63; 100
family	Character	NA	Taxonomic family	2,378	85	NA
genus	Character	NA	Taxonomic genus	2,378	233	NA
species	Character	NA	Taxonomic species	2,186	424^‡^	NA
habit	Integer	NA	Habit of N-fixing mosses and lichens code: if epiphyte or on the ground	213	2	1; 2
soil_depth	Numeric	cm	Soil depth sampled	152	18	0.5; 40
bulk_density	Numeric	g cm^-3^	Soil bulk density	13	8	0.00000241; 1
ref_code	Character	NA	Source code	1,529	376	NA
ref_complete	Character	NA	Complete source reference	376	376	NA

Detailed descriptions are in Supplementary Table S1. ^†^Number of unique pair of coordinates. ^‡^Number of unique species (*i.e*., Genus + species names). “NA” stands for not applicable.

### Data processing

Multiple entries in the dataset were either from the same administrative research areas or the same study sites within research areas. We harmonized these location names so that the same research areas and study sites have the same names across the dataset. We identified unique study sites as plots/stands that differed in vegetation composition or dominance, age, disturbance history, N fixer abundance, elevation/climate, and/or topoedaphic setting within the same research area. Although we identified individual study sites as plots/stands with different characteristics within research areas, these can be aggregated into larger spatial scales depending on the study goals (*e.g*., by research area or within a radius or grid cell). We filled gaps in geographic coordinates, vegetation type, N fixer or N-fixing substrate abundance, and soil bulk density using information gathered from sister publications by the same authors, provided directly by authors, or from other publications in the same study site/research area, whenever possible and appropriate.

We harmonized the vegetation type within and across study sites into one of the IGBP land cover classes^12^ (Supplementary Figure S1) based on the reported vegetation descriptions and definitions in the IGBP land cover system (Supplementary Table S2). For plantations of N-fixing species, we considered IGBP land cover classes where the N-fixing species naturally occur. For plantations of early-mid seral N-fixing species and plots/stands under ecological succession, we classified the land cover class as the corresponding late-successional vegetation type. We combined “Closed Shrublands” and “Open Shrublands” as “Shrublands” and combined “Woody Savannas” and “Savannas” as “Savannas” because most BNF studies lacked the necessary information for these finer classifications. We classified bogs and fens as “Permanent Wetlands” due to their water table being constantly close to the surface, and marshes as “Grasslands” and swamps as a forest biome, due to their more dynamic water table.

We standardized and updated family, genus, and species names in the dataset using The World Flora Online (WFO) database (http://www.worldfloraonline.org) and the Taxonomic Name Resolution Service (TNRS) online tool (https://tnrs.biendata.org)^16^ for species of trees, shrubs, herbs, mosses, and lichens. We checked names individually and used matched accepted names or accepted names converted from synonyms. For lichen species not listed in the WFO database, we checked for accepted names in the Consortium of Lichen Herbaria (https://lichenportal.org/portal/). We also used the National Center for Biotechnology Information (NCBI) (https://www.ncbi.nlm.nih.gov/taxonomy) and Algaebase (https://www.algaebase.org) taxonomy databases to check for accepted names of prokaryotes. Unresolved names (<0.1%) were kept as reported in the original publication.

**Fig. 3:**
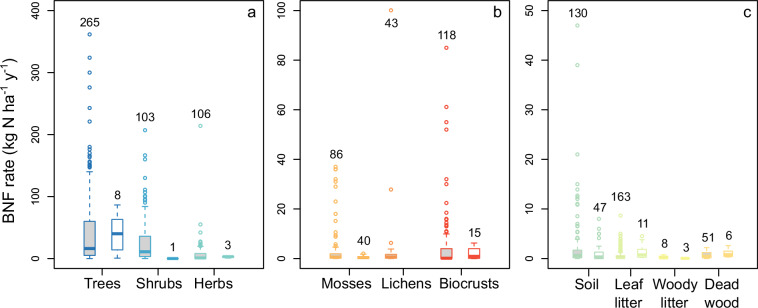
BNF rates originally reported in kg N ha^−1^ y^−1^ or similar units (grey filled boxplots) versus BNF rates converted to these units from cumulative or sub-annual BNF or ARA rates per unit of area or N fixer abundance, or %N_dfa_ values (white filled boxplots) for root-nodulating N-fixing trees, shrubs, and herbs (**a**), BNF associated with mosses, lichens, and biocrusts (**b**), and free-living BNF in soil, leaf litter, woody litter, and dead wood (**c**) in the global BNF dataset in natural terrestrial biomes^11^. Boxplots depict the median and interquartile range, with whiskers representing values within 1.5 times the interquartile range. Values outside this range are shown as individual data points. The sample sizes are indicated above each boxplot.

Published BNF rates were reported in 48 different units (Table 1). We provide BNF rates both in the reported units and standardized to kg N ha^−1^ y^−1^. The rates in the reported units are either the exact reported values or the mean or sum of reported group values. We averaged reported rates obtained using different methods or incubation conditions. We also averaged reported rates for replicates and seasonal or annual rates. We either summed or averaged rates to aggregate species-level into community-level rates. We summed species-level rates calculated using the local abundances of each species, while we averaged species-level rates calculated using 100% coverage (*e.g*., from samples or assumed). We also summed rates for different soil layers or depth intervals, and litter or dead wood classes or decomposition stages. The standardized BNF rates were either originally reported in kg N ha^−1^ y^−1^ or similar units (*e.g*., g N m^-2^ y^−1^) (n = 1,073) or were converted to these units from cumulative or sub-annual BNF or ARA rates per unit of area or N fixer abundance, or %N_dfa_ values (n = 134) (Fig. 3), as detailed below for each niche. All standardized BNF rates were adjusted to the local abundance of N fixers and N-fixing substrates, either by the authors in the original publication or in our analysis.

For ***trees***, we converted cumulative BNF rates across multiple years to mean annual rates by dividing by the number of years, though any calculation of mean rates might obscure interannual rate variation. We converted BNF rates per unit of N-fixing tree abundance to rates per unit of area using local N-fixing tree abundance data. We converted tree BNF rates per unit of nodule mass to rates per unit of area using local nodule biomass data for studies that measured both BNF activity and nodule biomass throughout the entire growing season. We also converted tree %N_dfa_ to BNF rates per area using local N fixer abundance and N content data.

For ***shrubs***, we converted cumulative BNF rates across multiple years to mean annual rates by division. When necessary, we converted shrub ARA rates per area to BNF rates using a conversion ratio of 3 mol of C_2_H_4_: 1 mol of N_2_ (R ratio)^17^. We used an R ratio of 3:1 for shrubs and other niches (see below) as it is the most applied theoretical ratio and is the single ratio that represents the majority of BNF rate data, even though the distribution of ^15^N_2_ calibrated R ratios from individual studies ranges widely above and below 3:1, which could affect individual BNF rates by 2 to >8 fold^18^. When available, direct calibrations provide more accurate results, so we used BNF rates estimated using direct calibrations rather than the assumed R ratio of 3:1 when presented in individual studies^18^. We also converted shrub sub-annual BNF or ARA rates to annual rates. We converted sub-annual ARA rates for studies that measured BNF activity at least twice during a growing season. We converted mean growing season rates to annual rates using 24 hours of activity per day and assumed growing season lengths (365 days for study sites located between latitudes 30N-30S, 180 days between 30–60 N/S, and 100 days between 60–90 N/S).

For ***herbs***, we converted ARA rates per area to BNF rates using an R ratio of 3:1^18^. We also converted herb sub-annual BNF to annual rates as described above for shrubs.

For ***soil***, we converted ARA to BNF rates using an R ratio of 3:1^18^. We converted ARA or BNF rates per unit of soil dry mass to rates per unit of area using local soil bulk density and applied these rates across the mean soil depth in our dataset (~ 10 cm, n = 152). If local soil bulk density was not available, we used the mean soil bulk density for the same biome type in the dataset. We also converted soil sub-annual ARA or BNF rates to annual rates as described above for other niches.

For ***litter***, ***woody litter***, and ***dead wood***, we converted ARA to BNF rates using an R ratio of 3:1^18^. We converted ARA or BNF rates per unit of dry mass to rates per unit of area using local biomass data. We also converted sub-annual ARA or BNF rates to annual rates as described above for other niches.

For ***mosses***, we converted ARA rates per unit of area to BNF rates using an R ratio of 3.3:1^19^. We did not use ARA rates for *Sphagnum* as these mosses exhibit a high abundance of methanotrophs, which can result in highly variable R ratios^20^. We also converted moss sub-annual ARA to annual rates as described above for other niches.

For ***biocrusts***, we converted ARA rates per unit of area to BNF rates using an R ratio of 3:1^18^. We also converted biocrust sub-annual ARA or BNF rates to annual rates using the mean number of days with precipitation >1 mm per year (average of 1991–2020) from CPC Global Unified Gauge-Based Analysis of Daily Precipitation provided by the NOAA PSL (https://psl.noaa.gov/data/gridded/data.cpc.globalprecip.html)^21,22^. We assumed 12 hours of BNF activity following a precipitation event for BNF measurements taken under light or dark conditions and 24 hours for measurements taken under both light and dark conditions.

We also calculated BNF rates per unit of N fixer abundance using the standardized BNF rates and reported local abundances of N fixers^8^ (Table 1). For trees, we calculated BNF rates per unit of N fixer relative basal area and/or N fixer relative stem density, *i.e*., the BNF rate (kg N ha^−1^ y^−1^) when N-fixing tree relative basal area is 1% (kg N ha^−1^ y^−1 ^%BA^−1^) and when N-fixing tree relative stem density is 1% (kg N ha^−1^ y^−1 ^%stems^−1^), respectively. For shrubs, mosses, and biocrusts, we calculated BNF rates per 1% N fixer ground cover (kg N ha^−1^ y^−1 ^%cover^−1^). BNF rates per unit of N fixer abundance are available for N-fixing herbs as %N_dfa_ values. BNF rates per unit of N fixer abundance indicate the additive increment for each 1% increase in N fixer abundance and allow scaling BNF rates per area using N fixer abundance data^8^. For example, for trees, 0.7 kg N ha^−1^ y^−^^1 ^%BA^−1^ yields 0.7 kg N ha^−1^ y^−1^ for 1% N-fixing tree relative basal area, 1.4 kg N ha^−1^ y^−1^ for 2% N-fixing tree relative basal area, and 7 kg N ha^−1^ y^−1^ for 10% N-fixing tree relative basal area.

For most niches, we provide species-level data for studies with one N-fixing species or community-level data for studies with more than one N-fixing species. For N-fixing herbs and mosses, we provide both species and community-level data, the latter weighted by the abundance of N fixers whenever species-level abundance data was available.

## Data Records

The dataset is available in the ScienceBase repository (10.5066/P1MFBVHK)^11^. It consists of 8 data files in the “.csv” format, each combining a subset of the variables (Table 1):

The “SITE.csv” file includes the variables study site name, ISO country code, geographical coordinates in decimal degrees, IGBP land cover class, N-fixing niche, ecological level (if species or community-level data), and the source code that links data to BNF study references in the “REFERENCES.csv” file, listed below.

The “BNF_AREA.csv” file includes the variables central, minimum, and maximum reported BNF rates and their unit, sampling year, and the quantification method(s) of BNF rates. This file also provides the variable final BNF rate in standardized units (kg N ha^−1^ y^−1^) and a variable that indicates whether final BNF rates were originally reported in kg N ha^−1^ y^−1^ or converted from similar units or other types of BNF data.

The “NDFA.csv” file provides the variables herb %N_dfa_, sampling year, and the quantification method(s) of %N_dfa_ values.

The “ABUNDANCE.csv” file includes the variables relative basal area and relative stem density for N-fixing trees, the percent ground cover for N-fixing shrubs, herbs, mosses, and biocrusts, and aboveground biomass for dead wood.

The “BNF_ABUNDANCE.csv” file provides the variables BNF rate per 1% N-fixing tree relative basal area, BNF rate per 1% N-fixing tree relative stem density, and BNF rate per 1% ground cover for N-fixing shrubs, herbs, mosses, and biocrusts.

The “SPECIES.csv” file lists the names of taxonomic families, genera, and species of N fixers.

The “AUXILIARY_DATA.csv” file has additional variables, including soil bulk density and sampled soil depth that supported rate conversions (see Data processing) and the habit of N-fixing mosses and lichens (if epiphyte or on the ground).

The “REFERENCES.csv” file lists the BNF study codes and complete references.

Except for the “REFERENCES.csv” file, all other data files include the variable “record identification code” linking data across files. Most files, namely “SITE.csv,” “BNF_AREA.csv,” “NDFA.csv,” “SPECIES.csv,” and “REFERENCES.csv” have individual variables as columns and observations as rows. The files “ABUNDANCE.csv,” “BNF_ABUNDANCE.csv,” and “AUXILIARY_DATA.csv” are “molten” files, combining variables into a single column and observations into another column^23^.

Detailed descriptions of all variables and values are in the accompanying “METADATA.csv” file and Supplementary Table S1. For manipulating the data using R programming language, see the accompanying “RCode_Data_manipulation_examples.txt” file. For manipulating the data using spreadsheet software, see Supplementary Text S2.

## Technical Validation

We reviewed and removed duplicate studies during the literature screening. If the same data were published in multiple formats, we selected data published as a journal article over other formats. We checked each data entry against its original publication at least twice, with particular attention to whether potential outliers represented data entry errors. We did not exclude potential outliers from the dataset as extreme values can represent hotspots or hot moments of BNF activity, which can account for a large proportion of BNF in natural ecosystems^5,24^.

**Fig. 4:**
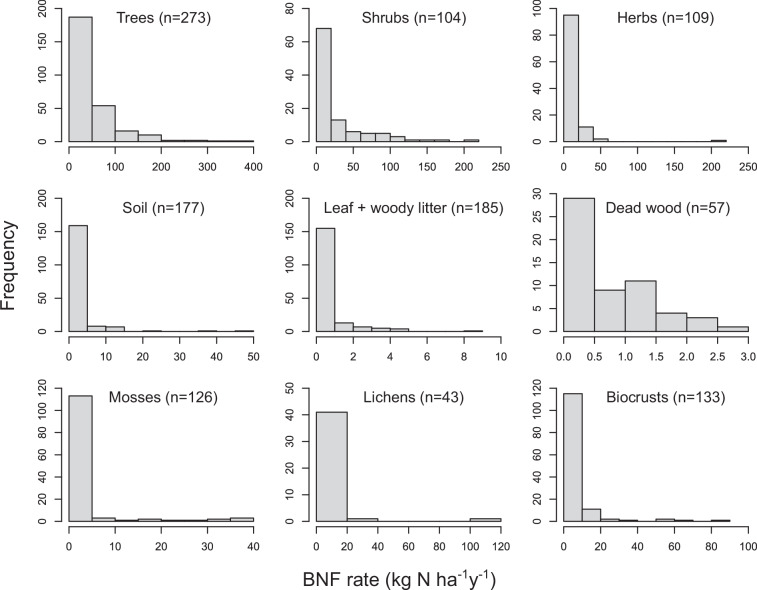
Frequency distribution of BNF rates (kg N ha^−1^ y^−1^) for root-nodulating N-fixing trees, shrubs, and herbs, free-living BNF in soil, leaf litter, woody litter, and dead wood, and BNF associated with mosses, lichens, and biocrusts in the global BNF dataset in natural terrestrial biomes^11^. The sample sizes are indicated in parentheses.

**Fig. 5:**
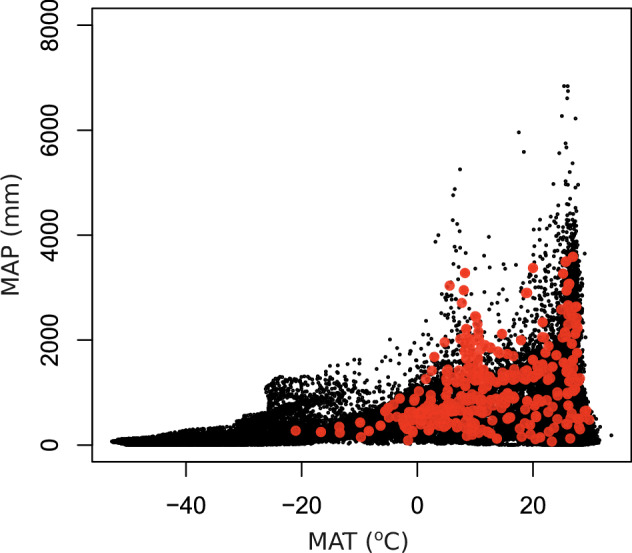
Climate space of study sites in the global BNF dataset^11^. The background black points represent the density of mean annual temperature (MAT) and mean annual precipitation (MAP) grid cell values at 0.004-degree resolution across natural terrestrial biomes from TerraClimate^26^ (average of 2000–2020), including areas permanently covered by snow or ice. Study sites in the global dataset are depicted as red points.

**Fig. 6:**
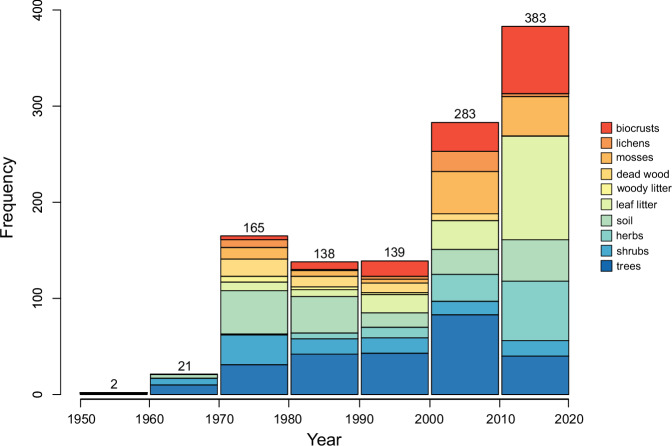
Frequency distribution of sampling year of BNF rates (kg N ha^−1^ y^−1^) for root-nodulating N-fixing trees, shrubs, and herbs, free-living BNF in soil, leaf litter, woody litter, and dead wood, and BNF associated with mosses, lichens, and biocrusts in the global BNF dataset in natural terrestrial biomes. Totals by decade are indicated above each bar.

We also checked BNF rate conversion calculations at least twice and enforced systematic procedures within and across niches, as described in Data Processing. All BNF rates converted from cumulative or sub-annual BNF or ARA rates per unit of area or N fixer abundance, or %N_dfa_ values, are within the range of BNF rates originally reported in kg N ha^−1^ y^−1^ or similar units (Fig. 3). The BNF rates in the dataset show a right-skewed frequency distribution across niches (Fig. 4), indicating frequent low and infrequent high values, typical of BNF fluxes^5,25^. The location of study sites in the dataset spans all continents but with a larger representation of mid-latitude areas in the Northern Hemisphere. Still, the location of study sites encompasses nearly the entire global range of temperature and precipitation (average of 2000–2020 from TerraClimate^26^) in natural terrestrial biomes (Fig. 5), except for extremely cold locations (MAT < −20 °C) where BNF is likely very low, and a handful of extremely wet locations (MAP > 4000 mm). The extensive coverage of Earth’s climate space suggests that the dataset is broadly representative of the heterogeneity of environmental conditions that drive BNF rates. Most of the BNF rates were measured between the years 2000 and 2020 (59%) (Fig. 6).

## Supplementary information

Supplementary Information

## Data Availability

Data visualization and manipulation were conducted using standard code packages in R version 4.3.0^27^. To facilitate user data manipulation, we provide accompanying R code with examples at 10.5066/P1MFBVHK^11^.
