# Supplementary material for: A global dataset of terrestrial biological nitrogen fixation
Source: Sci Data. 2025 Aug 5;12:1362. doi: 10.1038/s41597-025-05131-4 (PMC12325739; doi:10.1038/s41597-025-05131-4)
Supplement: Supplementary file 1 — Supplementary Information [file 41597_2025_5131_MOESM1_ESM.docx]

**Supplementary Information**

Reis Ely *et al.* A global dataset of terrestrial biological nitrogen fixation. *Sci Data*, 10.1038/s41597-025-05131-4 (2025).

**Contents**

Supplementary Text S1, page 1

Supplementary Text S2, page 2

Supplementary Table S1, page 4

Supplementary Table S2, page 7

Supplementary Figure S1, page 8

Supplementary Figure S2, page 9

Supplementary Figure S3, page 10

Supplementary Figure S4, page 11

Any use of trade, product, or firm names is for descriptive purposes only and does not imply endorsement by the U.S. Government.

**Supplementary Text S1.** Systematic Boolean search term.

The search term structure:

(N fixation terms) AND (terrestrial biomes list OR country list OR N-fixing taxa list OR N-fixing niches list)

("nitrogen fix*" OR "N2 fix*" OR "dinitrogen fix*" OR “N fix*” OR nitrogenase) AND ("temperate forest" OR “mixed forest” OR "tropical forest" OR “subtropical forest” OR rainforest OR “moist forest” OR “wet forest” OR “dry forest” OR arctic OR boreal OR tundra OR “high latitude” OR taiga OR subarctic OR grassland OR savanna OR desert OR shrubland OR dryland OR steppe OR meadow OR Cerrado OR Mediterranean OR fynbos OR chaparral OR matorral OR maquis OR kwongan OR “Antigua and Barbuda” OR “Bosnia and Herzegovina” OR “British Virgin Islands” OR “Burkina Faso” OR “Cabo Verde” OR “Cayman Islands” OR “Central African Republic” OR “Costa Rica” OR “Cote d’Ivoire” OR “Czech Republic” OR “Democratic Republic of the Congo” OR “Dominican Republic” OR “East Timor” OR “El Salvador” OR “Equatorial Guinea” OR “French Guiana” OR “Latin America” OR “Marshall Islands” OR “New Zealand” OR “North America” OR “North Macedonia” OR “Papua New Guinea” OR “Puerto Rico” OR “Saint Kitts and Nevis” OR “Saint Lucia” OR “Saint Vincent and the Grenadines” OR “San Marino” OR “Sao Tome and Principe” OR “Saudi Arabia” OR “Sierra Leone” OR “Solomon Islands” OR “South Africa” OR “South America” OR “South Sudan” OR “Sri Lanka” OR “State of Palestine” OR “Trinidad and Tobago” OR “Turks and Caicos” OR “U.S. Virgin Islands” OR “United Arab Emirates” OR “United Kingdom” OR “United States” OR Afghanistan OR Africa OR Alaska OR Albania OR Algeria OR Andorra OR Angola OR Anguilla OR Antarctica OR Argentina OR Armenia OR Aruba OR Asia OR Austria OR Australia OR Azerbaijan OR Bangladesh OR Bahamas OR Bahrain OR Barbados OR Belgium OR Belize OR Belarus OR Benin OR Bhutan OR Bolivia OR Botswana OR Brazil OR Brunei OR Burundi OR Bulgaria OR Cambodia OR Cameroon OR Canada OR Chad OR Chile OR China OR Colombia OR Comoros OR Congo OR Croatia OR Cuba OR Curacao OR Cyprus OR Djibouti OR Denmark OR Dominica OR Ecuador OR Egypt OR Eritrea OR Eswatini OR Estonia OR Ethiopia OR Europe OR Fiji OR Finland OR France OR Gabon OR Gambia OR Georgia OR Ghana OR Greece OR Greenland OR Grenada OR Guadeloupe OR Guatemala OR Guinea OR “Guinea-Bissau” OR Guyana OR Haiti OR Honduras OR Hungary OR Iceland OR India OR Indonesia OR Iran OR Iraq OR Ireland OR Israel OR Italy OR Jamaica OR Japan OR Jordan OR Kazakhstan OR Kenya OR Kiribati OR Korea OR Kuwait OR Kyrgyzstan OR Laos OR Latvia OR Lebanon OR Lesotho OR Liberia OR Libya OR Liechtenstein OR Lithuania OR Luxembourg OR Madagascar OR Malawi OR Malaysia OR Maldives OR Mali OR Malta OR Martinique OR Mauritania OR Mauritius OR Mexico OR Micronesia OR Moldova OR Monaco OR Mongolia OR Monserrat OR Montenegro OR Morocco OR Mozambique OR Myanmar OR Namibia OR Nauru OR Nepal OR Netherlands OR Nicaragua OR Niger OR Nigeria OR Norway OR Oman OR Palau OR Pakistan OR Panama OR Paraguay OR Peru OR Philippines OR Portugal OR Poland OR Qatar OR Romania OR Russia OR Rwanda OR Samoa OR Senegal OR Serbia OR Seychelles OR Singapore OR Slovenia OR Slovakia OR Somalia OR Spain OR Sudan OR Suriname OR Sweden OR Switzerland OR Syria OR Tajikistan OR Tanzania OR Thailand OR Togo OR Tunisia OR Turkey OR Turkmenistan OR Tuvalu OR Tonga OR Uganda OR UK OR Ukraine OR Uruguay OR US OR USA OR Uzbekistan OR Vanuatu OR Venezuela OR Vietnam OR Yemen OR Zambia OR Zimbabwe OR legum* OR Fabaceae OR actinorhizal OR Coriaria OR Datisca OR Alnus OR Allocasuarina OR Casuarina OR Ceuthostoma OR Gymnostoma OR Comptonia OR Myrica OR Morella OR Elaeagnus OR Hippophae OR Shepherida OR Colletia OR Discaria OR Kentrothamnus OR Retanilla OR Talguenea OR Trevoa OR Ochetophila OR Ceanothus OR Cercocarpus OR Chamaebatia OR Cowania OR Dryas OR Purshia OR Alder OR Dryas OR Sphagnum OR Hylocomium OR Pleurozium OR Ptilium OR Hylocomium OR Nephroma OR Peltigera OR Sticta OR Stereocaulon OR Collema OR Nostoc OR tree OR shrub OR scrub OR herb OR *lichen OR cyanobacteria OR moss OR liverwort OR free-living OR heterotroph* OR “soil crust” OR “biological soil crust” OR biocrust OR “cryptogamic crust” OR *symbio* OR cyanolichen OR endophyt*)

**Supplementary Text S2.** Data manipulation using spreadsheet software.

*Microsoft Excel*

We suggest using the Power Query tool for data manipulation in Microsoft Excel. To merge two different data files into a single spreadsheet, follow these steps:

1. Open the dataset files as separate sheets in the same Excel workbook.

2. Select the data in the first sheet, then go to the “Data” tab and click on the “From Table/Range” icon in the “Get and Transform Data” section to load the selected data into Power Query. Do the same for the second sheet.

3. In Power Query, select the first table, go to the “Home” tab, and click on “Merge Queries.” Select the second table for merging and the common column, “record_ID”, on each table. To keep all rows from the first sheet and add matching data from the second sheet, choose the “Left Outer” join kind. Other types of joins are also available.

4. Click on the small icon in the top right corner of the new column to expand and select which columns you want to add to your data. Then, click “Close & Load” to load the combined data back into Excel.

To merge more than two data files, merge two data files at a time.

Molten files (combining variables into a single column and observations into another column) must be reshaped before merging with other files. To do that, follow these steps:

1. Open the molten data file in Excel.

2. Select the data, then go to the “Data” tab and click on the “From Table/Range” icon in the “Get and Transform Data” section to load the selected data into Power Query.

3. In Power Query, select the “variable” column, then go to the “Transform” tab and click on “Pivot Column”. Select the “value” column.

4. Click on “Close & Load” to load the reshaped data back into Excel.

*Google Sheets*

To manipulate data using Google Sheets, use the function “query” to merge two data files:

1. Open the dataset files as separate sheets in the same Google Sheets file.

2. Create a blank sheet and copy and paste the “query” function below (adjust as needed):

=QUERY({Sheet1!A:B, Sheet2!B:C}, “SELECT Col1, Col2, Col3, Col4”, 1)

Where,

“{Sheet1!A:B, Sheet2!B:C}” combines data from Sheet1 columns A to B and Sheet2 columns B to C, with column A being the variable “record_ID” in both sheets,

“SELECT Col1, Col2, Col3, Col4” selects the columns to display in the result. Col1= “record_ID” in Sheet1, Col2= column B in Sheet1, Col3= column B in Sheet2, and Col4= column C in Sheet2,

and “1” indicates that the first row contains variable names.

The default of the “query” function is an inner join kind, that is, the result only includes rows with matching “record_ID” in both sheets. Other types of joins, as well as merging more than two sheets at once, are not as straightforward but possible using a combination of “QUERY”, “ARRAYFORMULA,” “IFERROR,” and “VLOOKUP” formulas.

To reshape molten data files before merging, follow these steps:

1. Open the molten data file in Google Sheets.

2. Select the data, then go to the “Insert” tab, click on “Pivot table” and create a new sheet.

3. In the Pivot table editor, select “record_ID” as rows, “variable” as columns, and “value” as values. By default, Google Sheets will also sum values in a separate column and row. Copy and paste the data, excluding summary statistics, into a new sheet.

**Supplementary Table S1.** Metadata of the global BNF dataset in natural terrestrial biomes.

| **File** | **Field** | **Description** | **Values** |
| --- | --- | --- | --- |
| SITE | record_ID | Record identification code that links data across files |  |
| SITE | site_name | Study site name. Similar site names followed by different numbers indicate plots/stands that differ in vegetation composition or dominance, age, disturbance history, N-fixer abundance, elevation/climate, and/or topoedaphic setting within the same study area |  |
| SITE | Country | 3-digit ISO country code | "Multiple" if data spans more than one country |
| SITE | Lat | Latitude coordinate in decimal degrees |  |
| SITE | Lon | Longitude coordinate in decimal degrees |  |
| SITE | IGBP | International Geosphere-Biosphere Program land cover class | "ENF" for Evergreen Needleleaf Forests, "EBF" for Evergreen Broadleaf Forests, "DBF" for Deciduous Broadleaf Forests, "MF" for Mixed Forests, "Shrub" for Closed Shrublands or Open Shrublands, "Sav" for Woody Savannas or Savannas, "Grass" for Grasslands, "Bare" for Barren, "Wet" for Permanent Wetlands, and "Snow" for Permanent Snow and Ice |
| SITE | Niche | N-fixing niche | "Trees", "Shrubs", "Herbs", "Soil" (mineral and/or organic soil, including humus), "Litter" (all non-woody litter, usually leaf litter), "Woody litter" (dead branches and stems ≤ 7.5 cm diameter), "Dead wood" (logs > 7.5 cm diameter), "Mosses", "Lichens", and "Biocrusts" |
| SITE | ecological_level | Ecological organization level the data refer to | "Species" or "Community" |
| SITE | ref_code | Source reference code in the form of "First author's last name_year of publication" |  |
| BNF_AREA | record_ID | Record identification code that links data across files |  |
| BNF_AREA | year | The calendar year that BNF was measured | The mean was entered if BNF was measured over multiple years |
| BNF_AREA | BNF_central | Central BNF rate reported | Value is exactly as reported, or the mean or sum of reported group values |
| BNF_AREA | BNF_min | Minimum BNF rate reported |  |
| BNF_AREA | BNF_max | Maximum BNF rate reported |  |
| BNF_AREA | BNF_unit | The unit BNF_central, BNF_min, and BNF_max are expressed |  |
| BNF_AREA | method | The method(s) used to quantify BNF rates | 10 = Acetylene Reduction Assay (ARA), 11 = ARA + ^15^N_2_ incorporation, 12 = ARA + N accretion, 13 = ARA + N accumulation, 20 = ^15^N_2_ incorporation, 21 = ^15^N_2_ incorporation + N accretion, 30 = ^15^N natural abundance, 31 = ^15^N natural abundance + ^15^N dilution, 32 = ^15^N natural abundance + N accretion, 40 = ^15^N dilution, 50 = N accretion, 60 = N accumulation, or 70 = Mass balance of other N fluxes |
| BNF_AREA | BNF_final | Final BNF rate in standardized units (kg N ha^-1^ y^-1^) based on BNF_central or BNF_min and BNF_max, and local N-fixer or N-fixing substrate abundance | If BNF_central is not available, BNF_final is based on the mean of BNF_min and BNF_max values |
| BNF_AREA | BNF_final_type | Indication on whether BNF_final was reported in the original publication, converted from similar temporal and/or spatial units, or converted from other types of BNF data. It also indicates whether rates were adjusted for local N-fixer or N-fixing substrate abundance in the original publication or here | 1 = reported in the original publication and already adjusted for local N-fixer or N-fixing substrate abundance, 2 = reported in the original publication and adjusted here for local N-fixer or N-fixing substrate abundance, 3 = converted from similar temporal and/or spatial units already adjusted for local N-fixer or N-fixing substrate abundance, 4 = converted from similar temporal and/or spatial units and adjusted here for local N-fixer or N-fixing substrate abundance, 5 = converted from other types of BNF data already adjusted for local N-fixer or N-fixing substrate abundance, or 6 = converted from other types of BNF data and adjusted here for local N-fixer or N-fixing substrate abundance |
| ABUNDANCE | record_ID | Record identification code that links data across files |  |
| ABUNDANCE | variable | N-fixer or N-fixing substrate abundance variable | "pct_BA" for N-fixer relative basal area (%BA), "pct_stems" for N-fixer relative stem density (%stems), "pct_cover" for N-fixer percent ground cover (%cover), or "AGB" for N-fixing substrate aboveground biomass (Mg ha^-1^) |
| ABUNDANCE | value | N-fixer or N-fixing substrate abundance value |  |
| BNF_ABUNDANCE | record_ID | Record identification code that links data across files |  |
| BNF_ABUNDANCE | variable | BNF per unit of N-fixer abundance variable | "BNF_BA" for BNF rate per unit of N-fixer relative basal area (kg N ha^-1^ y^-1^ %BA^-1^), "BNF_stems" for BNF rate per unit of N-fixer relative stem density (kg N ha^-1^ y^-1^ %stems^-1^), or "BNF_cover" for BNF rate per unit of N-fixer percent ground cover (kg N ha^-1^ y^-1^ %cover^-1^) |
| BNF_ABUNDANCE | value | BNF per unit of N-fixer abundance value |  |
| NDFA | record_ID | Record identification code that links data across files |  |
| NDFA | year | The calendar year that %N_dfa_ was measured | The mean was entered if %N_dfa_ was measured over multiple years |
| NDFA | method | The method(s) used to quantify %N_dfa_ | 30 = ^15^N natural abundance, 31 = ^15^N natural abundance + ^15^N dilution, or 40 = ^15^N dilution |
| NDFA | Ndfa | Percentage of nitrogen derived from atmosphere (%N_dfa_) |  |
| SPECIES | record_ID | Record identification code that links data across files |  |
| SPECIES | family | Taxonomic family |  |
| SPECIES | genus | Taxonomic genus |  |
| SPECIES | species | Taxonomic species | "sp" indicates one and "spp" indicates more than one unidentified species |
| AUXILIARY_DATA | record_ID | Record identification code that links data across files |  |
| AUXILIARY_DATA | variable | Auxiliary data variable | "habit" for the habit of sampled N-fixing mosses and lichens, "soil_depth" for the soil depth sampled for BNF (cm), or "bulk_density" for soil bulk density (g cm^-3^) |
| AUXILIARY_DATA | value | Auxiliary data value | 1 = on the ground, or 2 = epiphyte for N-fixer habit |
| REFERENCES | ref_code | Source reference code in the form of "First author last name_year of publication" |  |
| REFERENCES | ref_complete | Complete source reference |  |

**Supplementary Table S2.** The International Geosphere-Biosphere Program (IGBP) land cover classification system^12^.

| Land cover class | Description |
| --- | --- |
| Evergreen Needleleaf Forests | Dominated by evergreen conifer trees (canopy >2m). Tree cover >60% |
| Evergreen Broadleaf Forests | Dominated by evergreen broadleaf and palmate trees (canopy >2m). Tree cover >60% |
| Deciduous Needleleaf Forests | Dominated by deciduous needleleaf (larch) trees (canopy >2m). Tree cover >60% |
| Deciduous Broadleaf Forests | Dominated by deciduous broadleaf trees (canopy >2m). Tree cover >60% |
| Mixed Forests | Dominated by neither deciduous nor evergreen (40-60% of each) tree type (canopy >2m). Tree cover >60% |
| Closed Shrublands | Dominated by woody perennials (1-2m height) >60% cover |
| Open Shrublands | Dominated by woody perennials (1-2m height) 10-60% cover |
| Woody Savannas | Tree cover 30-60% (canopy >2m) |
| Savannas | Tree cover 10-30% (canopy >2m) |
| Grasslands | Dominated by herbaceous plants (<2m) |
| Permanent Wetlands | Permanently inundated lands with 30-60% water cover and >10% vegetated cover |
| Croplands | At least 60% of the area is cultivated cropland |
| Urban and Built-up lands | At least 30% impervious surface area, including building materials, asphalt, and vehicles |
| Cropland/Natural vegetation mosaics | Mosaics of small-scale cultivation 40-60% with natural tree, shrub, or herbaceous vegetation |
| Permanent Snow and Ice | At least 60% of the area is covered by snow and ice for at least 10 months of the year |
| Barren | At least 60% of the area is non-vegetated barren (sand, rock, soil) areas with less than 10% vegetation |


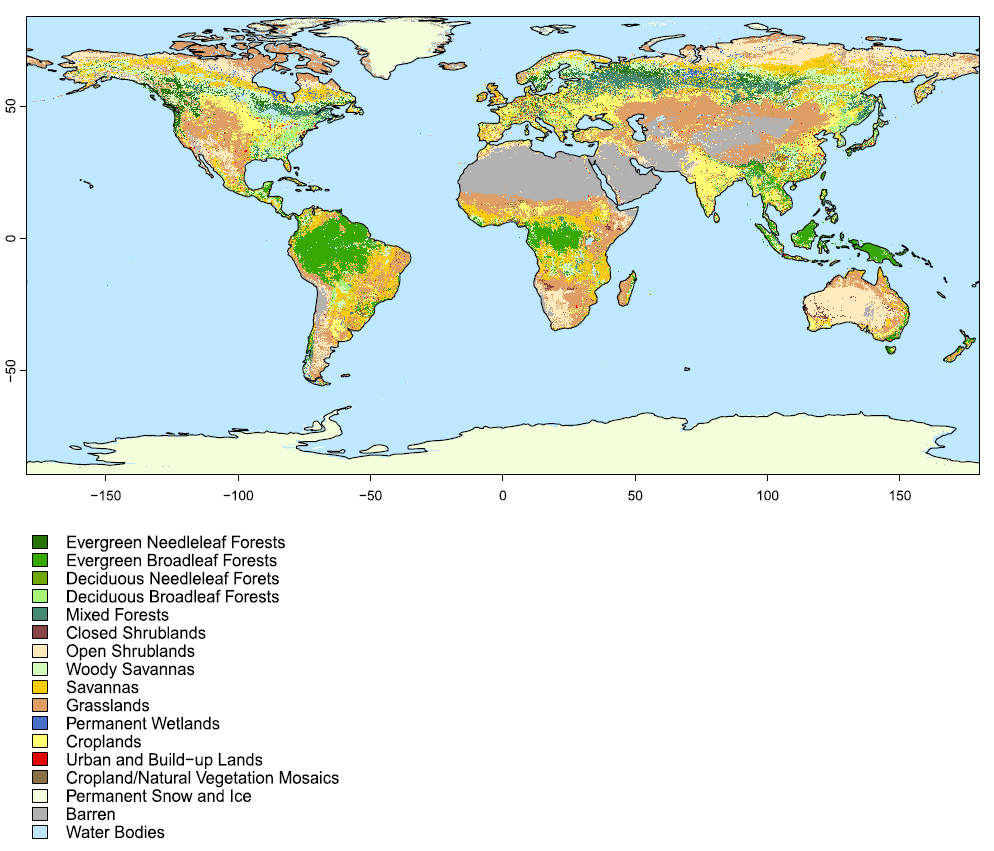


**Supplementary Figure S1.** The IGBP land cover classification system in 2019^12^.


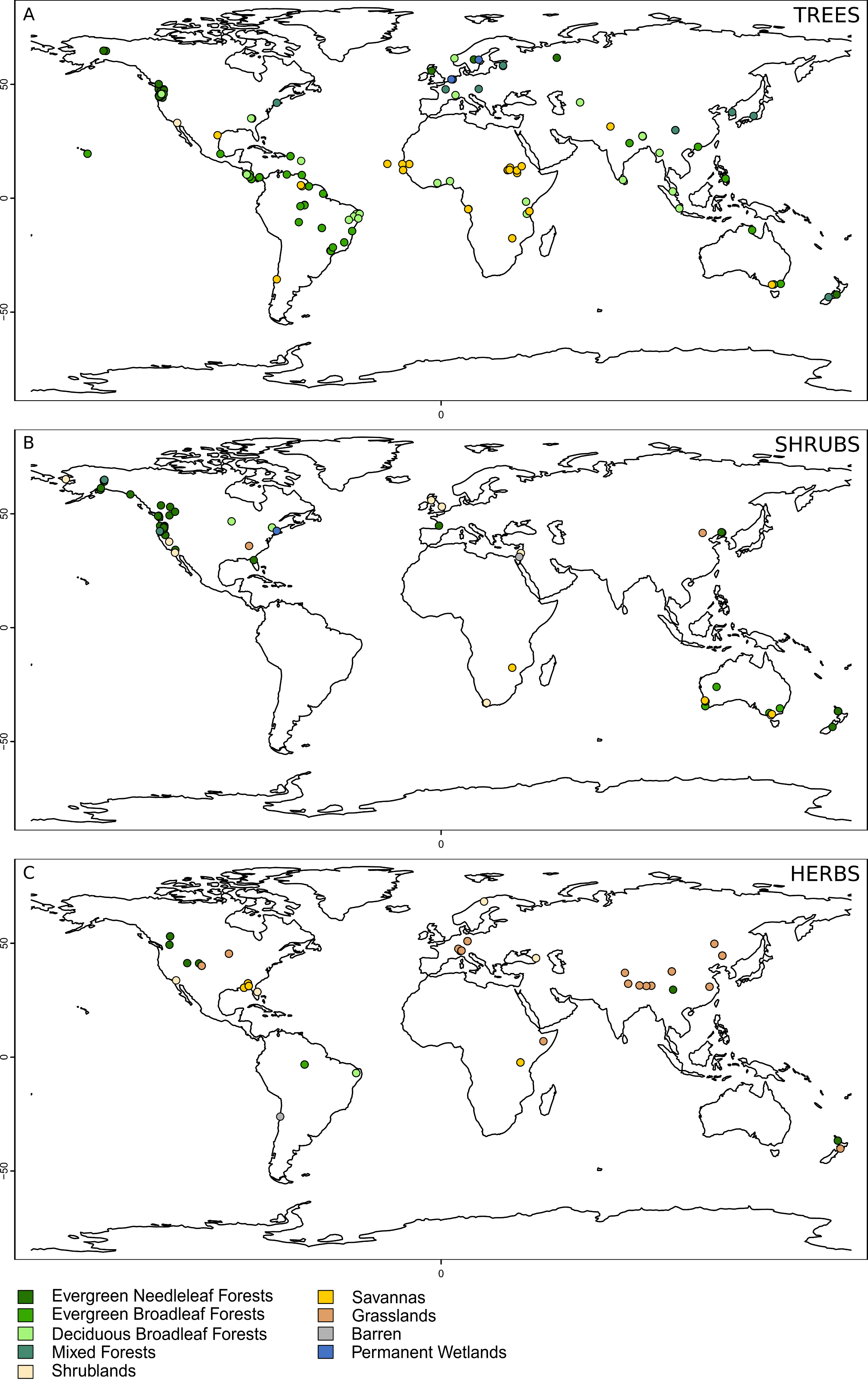


**Supplementary Figure S2.** Location of field measurements of BNF in natural terrestrial biomes for N-fixing trees (n=273) (A), shrubs (n=104) (B), and herbs (n=261) (C) by IGBP biome. Each point represents one BNF rate (kg N ha^-1^ y^-1^) and/or percentage of nitrogen derived from the atmosphere (%N_dfa_) value. Not all points are visible due to overlap.


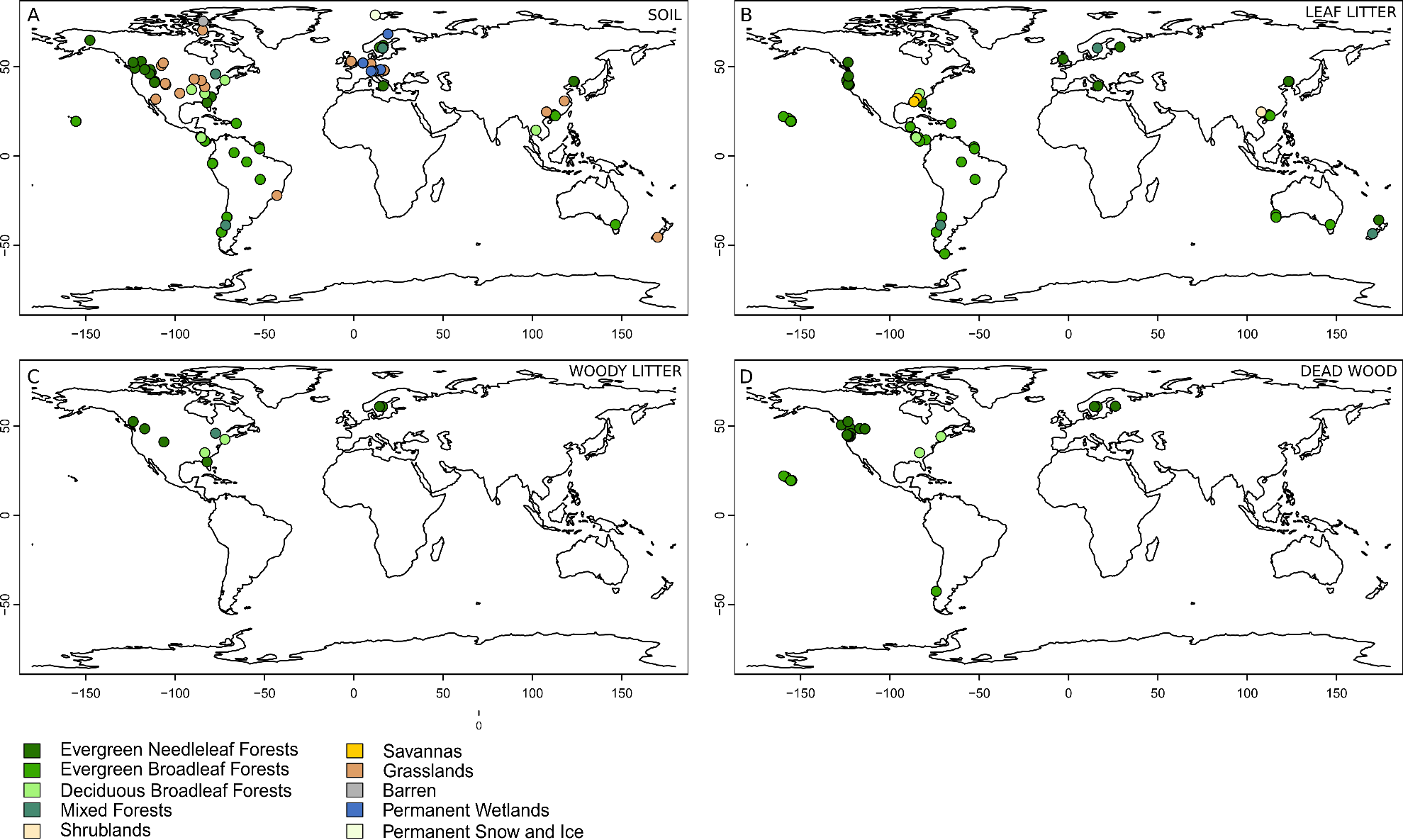


**Supplementary Figure S3.** Location of field measurements of BNF in natural terrestrial biomes in soil (n=177) (A), non-woody, mostly leaf litter (n=174) (B), woody litter (n=11) (C), and dead wood (n=57) (D) by IGBP biome. Each point represents one BNF rate (kg N ha^-1^ y^-1^). Not all points are visible due to overlap.


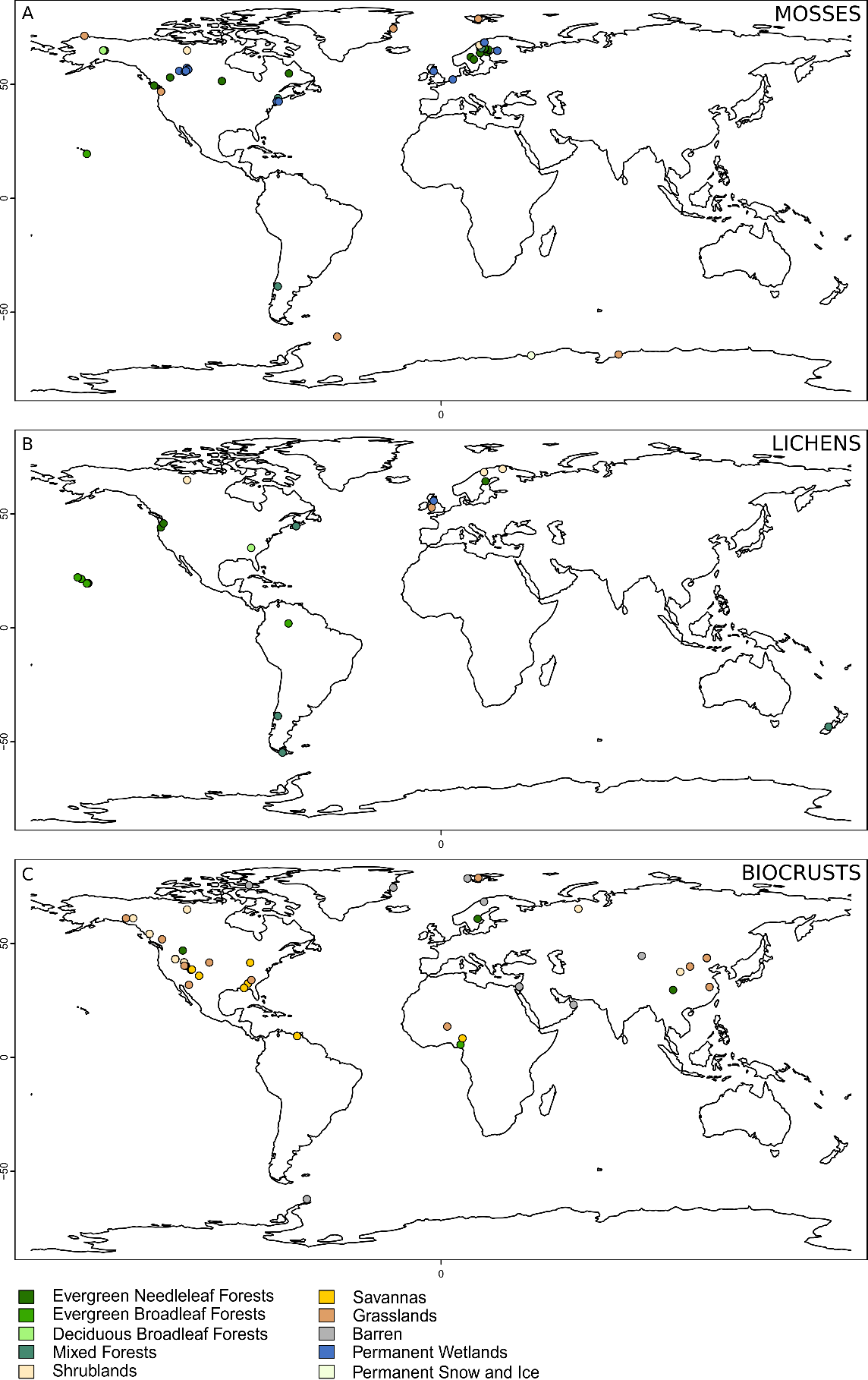


**Supplementary Figure S4.** Location of field measurements of BNF in natural terrestrial biomes for N-fixing mosses (n=126) (A), lichens (n=43) (B), and biocrusts (n=133) (C) by IGBP biome. Each point represents one BNF rate (kg N ha^-1^ y^-1^). Not all points are visible due to overlap.
